# The Use of Information and Communication Technologies in Family Support across Europe: A Narrative Review

**DOI:** 10.3390/ijerph19031488

**Published:** 2022-01-28

**Authors:** Ana Catarina Canário, Sonia Byrne, Nicole Creasey, Eliška Kodyšová, Burcu Kömürcü Akik, Aleksandra Lewandowska-Walter, Koraljka Modić Stanke, Ninoslava Pećnik, Patty Leijten

**Affiliations:** 1 Faculty of Psychology and Education Sciences, University of Porto, 4200-135 Porto, Portugal; 2 Department of Evolutionary and Educational Psychology, University of La Laguna, 38200 San Cristóbal de La Laguna, Spain; sbyrne@ull.edu.es; 3 Research Institute of Child Development and Education, University of Amsterdam, 1018 WS Amsterdam, The Netherlands; n.l.creasey@uva.nl (N.C.); p.leijten@uva.nl (P.L.); 4 APERIO, 12 800 Prague, Czech Republic; eliska.kodysova@aperio.cz; 5 Department of Psychology, Faculty of Languages and History-Geography, Ankara University, 06100 Ankara, Turkey; komurcu@ankara.edu.tr; 6 Institute of Psychology, University of Gdansk, 80-309 Gdansk, Poland; aleksandra.lewandowska-walter@ug.edu.pl; 7 Department of Social Work, Faculty of Law, University of Zagreb, 10000 Zagreb, Croatia; kmodicstanke@pravo.hr (K.M.S.); ninoslava.pecnik@pravo.hr (N.P.)

**Keywords:** information and communication technologies, ICT, family support, psychoeducation, online, self-directed programs, programs delivered through videoconference, professional-led support, peer-led support

## Abstract

The COVID-19 pandemic has accelerated the use of information and communication technology (ICT) to deliver parenting and mental health support services to families. This narrative review illustrates the diverse ways in which ICT is being used across Europe to provide family support to different populations. We distinguish between the use of ICT in professional-led and peer-led support and provide implementation examples from across Europe. We discuss the potential advantages and disadvantages of different ways of using ICT in family support and the main developments and challenges for the field more generally, guiding decision-making as to how to use ICT in family support, as well as critical reflections and future research on its merit.

## 1. Introduction

The COVID-19 pandemic has accelerated the provision of parenting and mental health support services to families online [1,2,3,4]. Organizations responsible for offering family support rapidly adjusted by implementing technology-based remote services [2], and several research teams adapted in-person parenting programs to be delivered remotely and evaluated their feasibility [5,6,7]. These adjustments ensured continued services to children and families and provided valuable insights into how service delivery methods can be transformed on a large scale [4,5].

Even though the application of information and communication technologies (ICT) accelerated during the pandemic, the merit of using ICT in family support has been studied for several decades [7,8]. The first two decades of the 2000s saw a rise in the use of technology in family support, with the aim of improving reach, access, and uptake and enhance the engagement of family support programs [9,10,11]. The most frequently studied form of technology-assisted family support is programs to improve parenting practices and reduce child mental health problems, delivered on desktop/laptop, tablet, or smartphone. Some of these interventions are fully self-guided, without therapist assistance (e.g., Triple P online [12]). Others include therapist feedback or phone calls (e.g., Parent–Child Interactions cellular enhanced version [13]) or are delivered by professionals through chat rooms (e.g., KopOpOuders in the Netherlands [14]) or WhatsApp (e.g., Parenting Education to Promote Parent–Infant Attachment in Turkey [15]).

The accumulated evidence shows that such technology-based parenting programs can successfully improve parent understanding of child development, parental feelings of self-efficacy, adaptive parenting behavior, and improve parent and child mental health [7,8,16,17,18,19,20,21]. Importantly, various studies suggest that parenting programs present similar results when delivered online as when delivered in person [5,8,17,22].

There are also other types of technology-based interventions described in the literature, including the use of videoconferencing, email consultation, and podcasts. Although these types are less thoroughly studied, there is some evidence to suggest that, following videoconferencing service delivery, parents reported improved parenting satisfaction and improved feelings of self-efficacy, whereas the clinicians revealed increased confidence and skills using technology [23]. Likewise, parents reported an increase in self-confidence following a single-session email consultation on the empowerment of parents [24]. Additionally, parents who had access to different podcasts on positive parenting over a period of two weeks, when compared to parents without access to the podcasts, reported improvements on measures of child behavioral problems and parenting style, self-efficacy, and confidence [25].

The purpose of this narrative review is to illustrate the diverse use of ICT in family support across Europe, presenting examples of current practices of professional-led and peer-led support that are available for families and that go beyond what has been evaluated in empirical studies. This work represents an output of the European Union Cost Action “The European Family Support Network: A bottom-up, evidence-based, and multidisciplinary approach” (CA18123, EurofamNet, https://eurofamnet.eu/, accessed on 10 November 2021) [26].

The Action report by Devaney and colleagues [27] conceptualizes family support as a set of activities implemented or delivered to improve family functioning and child rearing and other types of family activities in a system of supportive relationships and resources (formal and informal). A strong emphasis is put on a child-protection perspective, through which family support “involves a set of activities and access to practice that encourages positive informal social networks through integrated programmes which combine the statutory, voluntary and private agencies and services” (p. 13) [28]. Family support activities are provided by a workforce which delivers emotional advice and esteem support to families. Professionals and paraprofessionals (e.g., volunteers and interns) from multiple areas and from the government or non-governmental organizations organize, provide, and advocate for services within the human and child rights framework, supporting different aspects of family functioning and including a family support approach in their daily practice [29].

The Action report also acknowledges that family support is defined in different ways in different contexts. Family support is described as a multidisciplinary response to intervention and prevention of risk which emphasizes the importance of social networks and where the promotion of children’s well-being is central [27]. Parenting support, as part of family support, includes different activities and services that are provided with the purpose of “improving how parents approach their role as parents and to increasing parents’ child-rearing resources (including information, knowledge, skills and social support) and competencies” (p. 12) [30].

This narrative review followed a non-standardized methodology where the search strategies, comprehensiveness, and time range varied and did not follow an established protocol. We opted for this approach, rather than for a systematic literature review, for three main reasons: (1) diversity: limiting the search to the effectiveness of online family support would miss other, less intensively studied resources available for families, such as websites, apps, or peer-led support activities; (2) coverage: we aimed to include examples from all over Europe, including work from countries that may be underrepresented in the international literature; and (3) timing: some of the examples provided are very new, and a literature search of published work would miss this ongoing work.

First, we gathered examples provided by the participants (i.e., researchers and stakeholders) enrolled in the Action’s Working Group 3 (WG3) on quality standards and evidence-based family support programs. The participants in WG3 primarily work with parenting interventions targeting parents of younger children, and this is one of the reasons why most of the examples provided regard parenting interventions addressing parents of children up to school age. Second, we conducted searches in scientific and grey literatures and in general search engines (e.g., Google) and social media websites (e.g., Facebook). These searches were run in several languages (Croatian, Czech, Dutch, English, Polish, Portuguese, Spanish, and Turkish), and no time limit was set. For example, to identify examples of self-directed online programs and support through video calling, we searched, in addition to Google and Google Scholar, Academic Search Ultimate, Fonte Acadêmica, APA PsycArticles, APA PsycINFO, Psychology and Behavioral Sciences Collection, Sociology Source Ultimate, Scopus, and Web of Science using the search terms (1) family or parenting AND intervention AND online or self-directed, and (2) family or parenting AND support or interventions or programs AND parents of children and/or adolescents AND delivered through video call or videoconference or telehealth AND Europe. Third, we used the reference lists of systematic reviews and meta-analyses on this topic [10,20,21].

In the next section we describe the ways in which ICT can be used in family support, through professional and peer-led support. For each use of ICT in family support, we briefly explain the approach, provide examples of its implementation across Europe, and discuss available evidence of benefits and/or possible disadvantages for different populations.

## 2. Ways in Which ICT Can Be Used in Family Support

### 2.1. Professional-Led Support

Professional-led support includes the materials and resources that are made available by professionals through ICT to support families. These often include psychoeducational websites, self-directed online resources, and family support through videoconference. Most professional-led, online family support programs are parenting programs that include information on positive parenting (e.g., positive involvement and descriptive praise), proactive parenting (e.g., setting rules and limits), and relationship building (e.g., play and other activities to improve the quality of parent–child interactions) [12,31,32,33,34,35]. This information is very similar to parenting programs offered in person, although the delivery of the information may differ [36].

#### 2.1.1. Psychoeducational Websites and Apps

Psychoeducation is an intervention that combines the didactic transfer of information with emotion processing and motivational techniques to support individuals or families in addressing challenges and developing coping skills [37]. Psychoeducational family support websites typically entail written, audio, and video material with information on topics relevant for families (e.g., (a)typical child development, parenting difficulties) and suggestions on how to address difficulties or concerns (e.g., adjust parental expectations, behavior management techniques). Some psychoeducational family support websites also include a message board where parents can, anonymously or not, post conversational topics, questions, or any other information they might want to share [38].

There are several psychoeducational websites across Europe that provide information on child development and family support with content developed by professionals. One example is the Spanish website http://www.familiasenpositivo.org/ (accessed on 11 November 2021), endorsed by the Spanish government with the aim of promoting and supporting positive parenting by providing citizens with relevant information and directing them to available resources across the country [39]. Another example is the website regarding support for parents developed by the UK charity NSPCC, https://www.nspcc.org.uk/keeping-children-safe/support-for-parents/ (accessed on 11 November 2021), which includes parenting tips for all stages of children’s development, parenting advice on how to deal with difficult situations, a positive parenting guide (available in English, Welsh, French, and Arabic), a direct connection to a helpline, and links to further resources related to family support [40].

There are also professional Facebook pages such as Parenting for Lifelong Health [41], providing open-source and evidence-based resources to support parents and caregivers during COVID-19 and beyond. The resources focus on building positive parent–child relationships and reducing violence against children by learning through play, reinforcing positive and managing difficult behaviors, creating structure and routines, talking about COVID-19, keeping children safe online, and reducing stress and conflict in the family. The page works, among others, with the WHO, UNICEF, the Global Partnership to End Violence against Children, and the University of Oxford. The resources are available in different languages. The Parenting for Lifelong Health Programs are currently being implemented in several countries across Europe, including the Czech Republic, Moldova, Montenegro, North Macedonia, and Romania.

An example of a psychoeducational family support app is the UNICEF ECARO “Parent Buddy” app [42]. This app was developed to reach and support parents of children aged 0 to 6 years, with comprehensive, evidence-based information and interactive tools to cover many aspects of children’s health and development. Parent Buddy was developed to provide parents with customized guidance according to the needs and characteristics of their children. It also includes a library of articles and videos grouped into themes that include play and learning, safety and protection, and responsive parenting, among others. ECARO developed the app in partnership with UNICEF Serbia and the City Institute of Public Health, Belgrade. The app is currently in phase 2, optimization, which includes the customization of the app for launching in 10 countries: Albania, Bulgaria, Greece, Kosovo, Montenegro, North Macedonia, and Serbia in Europe, and Kyrgyzstan, Tajikistan, and Uzbekistan in Central Asia. The next phase, deployment, is where the app is intended to be scaled up in Europe and Central Asia and is expected to be completed in 2022.

The advantage of psychoeducational websites and apps as resources to support families is their availability to many families. These resources are, therefore, useful to deliver universal information on family support and to inform families about how to access more specialized support in their area should it be needed. Most psychoeducational websites, thus, mainly allow families the opportunity to gain knowledge or obtain information, rather than providing support in the form of specialized interventions.

In terms of the benefits of psychoeducational websites, a meta-analysis of 12 studies found that web-based programs have, indeed, contributed to improvements in parental knowledge, behavior, and attitude [20]. Effects seemed to be more favorable for psychoeducational websites addressing a specific issue than for websites with a broad, public health orientation on daily parenting.

#### 2.1.2. Self-Directed Online Programs

A more active form of professional-led, online support includes self-directed, online parenting programs. Many parenting programs offered online are adapted versions of programs originally designed to be offered in in-person settings. This means that the content is generally similar but delivered in a different format. This format typically includes less interaction with professionals or other parents. Instead, many programs use written materials, videos, and assignments for practice and self-evaluation [32,43]. However, programs increasingly use more diverse delivery methods, such as portals that allow professionals to tailor content to parents’ online activities [44], or combine online programs with professional-led phone calls [34].

In one of the first European evaluation trials of online parenting programs, in Sweden, Enebrink and colleagues [32] tested the effects of an online version of Comet, a social learning theory and cognitive behavior theory program [45]. The program consisted of seven sessions offered on a secure website with written text, videos of parent–child interactions, and illustrations. Relative to a waitlist control, the program reduced parent-reported child behavior problems and increased parent-reported child prosocial behavior. Reductions in behavior problems were strongest in families where parents completed more sessions.

Online parenting programs might be a valuable alternative to parents for whom it is more difficult to attend programs in person. Indeed, attendance and engagement rates in online programs tend to be relatively high. In Spain, for example, ‘Educar en Positivo’ showed response rates higher than those typically seen in in-person parenting programs [46]. Several meta-analyses suggest that online parenting programs can positively change family dynamics and child mental health, but the evidence for online programs is, so far, less robust than that of in-person programs [21,47]. Not only is the number of independent evaluations still relatively small, but the majority of online parenting program evaluation studies relied solely on parent reports and lacked measures that are arguably less subjective (e.g., video-taped parent–child interactions or teacher reports of child mental health).

Other examples of self-directed online programs are (1) the web-assisted self-help (WASH) for parents of children with attention-deficit/hyperactivity disorder (ADHD) [48], an intervention available in Germany to support parents of school-aged children with ADHD or other externalizing behavior problems; and (2) the online program for divorcing families, Family Doesn’t End with a Breakup, available in the Czech Republic [49,50].

Several individual studies and meta-analyses tried to identify program characteristics that enhance the effectiveness of online parenting programs. For example, some studies suggested that adding professional contact to online programs increases their effects [10,13], and a meta-analysis of the effects of online programs suggested that sending parents reminders to work on the program is associated with stronger effects [21]. Here, too, however, it is important to note that sound, well-powered evaluations comparing different types of online parenting programs are limited.

#### 2.1.3. Family Support through Video Calling

A third form of professional-led, online family support is through the use of video calling, also known as videoconferencing. Parenting programs can be delivered to parents in their homes through videoconferencing platforms (e.g., Zoom or Skype) using a device such as a smartphone, tablet, or a computer. This allows the professional to interact with one or more families in real-time using therapeutic techniques from the in-person program, such as motivational interviewing, video feedback, group interaction, and role play. Several evidence-based parenting programs have been formerly adapted to include videoconferencing within a telehealth delivery model (e.g., Incredible Years, Triple P, and Family Check-Up), while retaining the core components of the in-person program (e.g., positive behavior support). Notably, based on the literature, most telehealth delivery models of parenting programs have coupled live videoconferencing with additional online education modules that parents complete independently at home. However, more recently, research has emerged testing the delivery of parenting programs exclusively through videoconferencing.

For example, the Group Lifestyle Triple P (GLTP) was delivered to families in Portugal through the platform Zoom during the COVID-19 pandemic, with results similar to those found in studies of the in-person program [6]. The GLTP is a group format, evidence-based parenting intervention that combines components of positive parenting with advice to promote a healthy lifestyle in order to tackle childhood obesity [51]. Canário and colleagues made several minor adaptations to the delivery of the group sessions to enable delivery using videoconferencing. For example, the ‘breakout room’ option in Zoom was used for parents to complete tasks in small groups (e.g., practicing praising your child) and, in some instances, PowerPoint slides containing pictures (e.g., food labels) were sent to the parents in advance of the session for them to use during the group tasks. For one task, as an alternative to a group role play, parents were also asked to video record themselves playing an indoor game with their child that they then shared with other parents during the session. Another adaptation included replacing some tasks with presentations by the group facilitator, which included showing video examples of parent–child interactions. According to the authors, these adaptations did not compromise the fidelity of the program, and the group facilitator evaluated the experience positively. Moreover, participant retention (87.50%) and attendance (92.86%) were high, and parents reported high levels of satisfaction in both a post-intervention questionnaire and interview. Interestingly, the group facilitator highlighted that the creation of a WhatsApp group for one of the tasks was a particularly positive adaptation that fostered each family’s social support network [6]. As such, it may be particularly fruitful for practitioners to consider combining delivery of parenting programs using videoconferencing with other social platforms that allow interaction between families and facilitators.

As a second example, in Croatia, the Growing Up Together Centre for Parenting Support has developed technology-assisted delivery of two programs: Growing Up Together – Online and Growing Up Together in a New Family. Growing Up Together–Online [52] targets parents of children under the age of 6, who raise their children in adverse parenting and life circumstances (e.g., low income, single parents, survivors of domestic violence) and use social welfare and/or are engaged with the child protection services. Parents are referred to the program from social services (family support centers/family mediation and counseling or social welfare/child protection centers). The purpose of the program is to enable the exchange of information, knowledge, skills, and support around parenting, as well as promote personal growth and the development of parent and child competencies within the context of the pandemic. It was designed as a hybrid model that combines an in-person and a remote approach; the former referring to the first and the final workshop intended for the parents and their children to participate in together, and the latter referring to the eight thematic workshops in between that were intended only for parents to participate in using tablets provided by the center. Growing Up Together in a New Family [53] targets adoptive parents and their children (aged 2 to 10) and consists of 12 workshops designed to have several modes of delivery: (1) full in-person (in small groups), (2) full online—via videoconferencing (in small groups), and (3) full online—via interactive network resources (individually). Each workshop combines several techniques (short lectures with PowerPoint presentations, video content, different modes of group and individual work) that alternate with group discussion.

As a third example, in Italy, professionals from the Child Neurology and Psychiatry Unit of the IRCCS Mondino Foundation in Pavia developed Engaging with Families in Online Rehabilitation of Children (EnFORCE) during the COVID-19 pandemic [54]. The program targets families with children with special health care needs and uses tailored sessions through videoconference to offer parental support and child rehabilitation. Parental support includes parent education sessions where professionals provide parents with materials and strategies to improve parent–child activities at home and support sessions where professionals actively listen to parents’ daily challenges and provide emotional support. The EnFORCE program was first implemented in Italy during the first COVID-19 lockdown in 2020, with a sample of 36 families. The majority of families reported increased feelings of engagement, perceived support, and recognition of their role in childcare at the end of the intervention [54].

Other examples of support delivered through videoconference include online support for parents of gifted children in Turkey [55] and professional-led support groups for parents who want to strengthen their parenting competences, better understand themselves and their child, and prepare solutions for emerging difficulties [56] and for parents of teenagers with mental health problems [57].

Delivering parenting programs and other forms of family support by videoconferencing may be particularly convenient for families that find it difficult to visit a provider in person, for example, due to work commitments or difficulties arranging childcare. Moreover, videoconferencing may also reduce burden on practitioners by reducing the time needed to travel to the site of program delivery (e.g., a family’s home or a space suitable for group work). Potentially, by reducing logistical difficulties on the part of both the parent and practitioner, delivery by videoconferencing could improve accessibility and increase retention of families to parenting programs. However, it is important to bear in mind that, when using videoconferencing technology, as well as other types of ICT, the ability to use such technology, a stable internet connection, and/or a quiet space in which to take a video call or take part in a self-directed program may not be available to all parents. In particular, socially disadvantaged families may have difficulty accessing the resources required to take part in programs offered via videoconferencing or online; therefore, it is essential that we continue to offer programs in multiple formats.

Delivery of family support programs by videoconferencing potentially shares some of the benefits that have previously been identified for telephone delivery of family-based psychotherapy; in particular, aiding understanding of the home environment, reducing the stigma associated with receiving support, and being able to establish a convenient schedule [7]. On the other hand, a key consideration when using videoconferencing is whether the lack of in-person contact compromises the therapeutic alliance and/or group dynamics, which could potentially compromise intervention effectiveness. Encouragingly, a recent review synthesizing findings from 14 clinical trials of telepsychotherapy suggested that a strong therapeutic alliance can be maintained using videoconferencing [7], which aligns with other research indicating that in-person contact is not necessary for therapeutic alliance [58,59]. As for group dynamics, videoconferencing can restrict the ability to partake in group tasks, such as role plays, which may inhibit the connection between families. However, in the aforementioned study of GLTP delivered by videoconferencing, the facilitator evaluated the group dynamic positively and parents reported that social support gained from the group experience was a significant aspect of the program for them [6].

### 2.2. Peer-Led Support

Whereas the vast majority of the literature on online family support has focused on professional-led websites and programs, most families using online family support may use online support from peers (i.e., other families) [60]. Online peer-to-peer support is a form of support in which, by using online platforms, users can obtain information and support that is important to them, as well as provide assistance to other users [61]. Although online peer support might intuitively be associated with the ‘social media era’ for many, peers have been supporting each other in a wide range of informal, experience-based internet forums since the 1980s. Peer support is often initiated by parents with specific experiences, such as having a child with a mental or physical health problem, and offers a social network for receiving and giving emotional support and advice among others who share similar experiences, without geographical barriers [62].

There are different forms of online peer support: asynchronous and synchronous [63]. Asynchronous forms include, for example, internet groups, forums, or message boards where users can share important information, ask questions, and answer others’ questions. Synchronous forms, on the other hand, allow for constant contact with the online person on duty or with another member of the group who is currently online (e.g., chat rooms). Online support can be open to the public or it can be closed, whereby only a select group of users are admitted, which may or may not change over time [64].

Popular examples of peer-led groups that offer support to families include Facebook/Instagram groups and instant messaging groups (e.g., WhatsApp/Telegram groups). One European example is the UK Facebook group ‘Tots Together’, with 42,439 followers [65], creating content on parenting and lifestyle. For example, the profile offers a free, online baby group three days a week for caregivers and children who are in need of support for whatever reason, such as feeling depressed, struggling with stress, or experiencing loneliness. Whether or not they participate in the online group, followers have the opportunity to share their feelings and thoughts under such a post, allowing them an emotional outlet and a way to engage others’ support.

Several factors increase the likelihood that parents engage in online, peer-to peer support: (1) limited access to the traditional support networks, (2) a feeling of living with the stigma of illness, (3) belief in their own similarity with other users and people providing support, (4) the convenience of using modern technologies to communicate, and (5) willingness to remain anonymous or preference to share with strangers [64,66].

Research confirms many advantages of using online, peer-led support, including an increase in the level of perceived support, re-education of the feeling of exclusion and isolation, a decrease in the feeling of being stigmatized, a sense of connection with people experiencing similar problems, and better coping with everyday problems [61,67,68,69]. Several challenges are identified regarding online, peer-led support, with one of the most relevant being to ensure the transmission of reliable information. The lack of a moderator, specifically someone with experience in family support, may lead users to provide each other with unverified information or misinterpret information that is available on the internet. Online interactions can also reduce the importance of real relationships and contact with loved ones, which are important in the process of recovery and coping with stress. As a consequence, a person who excessively uses the internet and online support may tend to avoid in-person contact [61,70,71,72].

## 3. Discussion

The purpose of this narrative review was to illustrate the diverse ways in which ICT is used in family support across Europe, their main characteristics, and evidence for their benefits and possible disadvantages. We included various examples of professional- and peer-led support across Europe, of which some are relatively new or rarely evaluated in international literature. The examples show that there are many advantages to delivering family support through ICT, as opposed to delivering family support only in person. The advantages include: flexible scheduling, according to the families’ routines and work commitments; and enhanced accessibility, including engaging underserved families, enabling some family members to participate who otherwise might have not been able to, and improving access to families living in remote areas or lacking transport to attend in-person interventions [3,4,11].

Whether delivering family support through ICT should be preferred over delivering family support in person, depends on various factors. For example, many of the examples discussed in this review do not include active professional involvement. Psychoeducational websites, for example, present information but cannot check whether parents understand this information and do not allow them to ask questions. Similarly, advice provided in peer-led discussion groups that are not moderated by a professional may be ineffective or even harmful. Several types of ICT-based family support may, therefore, be less suitable for families at risk of problems and in need of more specialized services [11]. These families might benefit from other ICT-based family support, such as videoconferencing, and from in-person support. In addition, families need to have access to resources that allow them to engage (e.g., devices, internet, literacy, and technology proficiency). Importantly, however, while some suggest that it is more challenging to reach families with fewer resources for family support through ICT [4], a recent meta-analysis supported the use of family support through videoconferencing for families experiencing social disadvantage [10]. Even in families with little to no computer literacy, professionals and families can engage in technology-based family support with some patience and support from the professional [7]. In fact, the non-governmental organization SOS Children’s Villages has been committed to using ICT for development, through ICT4D – Technology for children, young people and families [73]. By doing so, the NGO expects to provide access to the internet and digital technologies and enhance digital literacy among vulnerable children, young people, and families; thus, broadening their development opportunities.

That said, various challenges remain for optimal use of ICT in family support, in addition to the general notion that more passive types of family support may not be suitable for those needing specialized services. First, not all families may have access to devices and internet but, due to home-schooling during the pandemic, many governments distributed resources (e.g., tablets, computers) to socioeconomically disadvantaged families, and internet became more affordable in many countries. For example, Lithuania and Croatia offer free wi-fi services to locals, and some countries (e.g., Portugal) have introduced lower internet prices for socioeconomically disadvantaged families.

Second, there are important ethical issues that must be considered when using online services. Without the presence of a professional, it can be questioned whether the parent can have any questions about the service fully answered and provide informed consent which might affect their decision on the best service for their family’s needs. Other examples include confidentiality and data protection, which can be compromised when using an internet connection [1,11]. Potential ways in which confidentiality could be compromised include other parties accessing the live sessions and session recordings/chat logs being stored by other parties. However, there are several ways these issues can be overcome; for example, (1) restricting session access using passwords, (2) turning on a waiting room function to give the facilitator control over who enters the session, (3) using a professional account with software approved by one’s institution or organization, and (4) obtaining family consent and making a data management plan for any recorded data collected during sessions. Last, although digital/technological contact with professionals and peers can complement a lack of traditional social support [74], there are reasons to question whether it can fully replace in-person social contact and support. Increased emphasis on the use of ICT in family support might, thus, come at the cost of in-person support [68,75].

Next steps for the field include improving our understanding of the quality of resources available to families and determining whether they are in fact helpful. While more traditional, online parenting programs have been evaluated in rigorous trials, exposure to some other types of ICT-based family support (e.g., peer-led WhatsApp groups) have not. This is especially important because some of these platforms are being widely used by families [60]. In addition, a shift is needed in research comparing the relative effects of ICT-based versus in-person family support, to research and identify the conditions under which certain families benefit more from different delivery types. Methodological developments, such as the personal advantage index [76], allow for secondary analyses of studies comparing different intervention types and increase our ability to predict which families benefit more from each intervention type.

The examples presented in this narrative review are drawn from the experience of researchers and stakeholders participating in EurofamNet WG3, complemented by the results of various, non-systematic literature searches. The advantage of this is that we were able to include examples that are not included in the international academic literature. The disadvantage of this is that the examples are selective (e.g., mainly on parenting support targeting parents of younger children) and do not present a complete picture of the use of ICT in family support services in all European countries. Further research is warranted to address such limitations.

## 4. Conclusions

This narrative review illustrates the diverse ways in which ICT is being used to offer family support across Europe. Benefits of the recent increase in the use of ICT include the potential to reach more families, especially when combined with national developments to increase families’ access to devices and the internet. In line with the goals of a narrative review, we did not set out to cover all the ways in which ICT is being used and did not define inclusion and exclusion criteria. We hope our review can inspire and guide decision-making as to how to use ICT in family support, as well as critical reflections and future research on its merit.

## References

[1] Burbach F., Pote H. (2021). Digital approaches—A paradigm shift?. J. Fam. Ther..

[2] Gurwitch R.H., Salem H., Nelson M.M., Comer J.S. (2020). Leveraging Parent-Child Interaction Therapy and telehealth capacities to address the unique needs of young children during the COVID-19 public health crisis. Psychol. Trauma Theory Res. Pract. Policy.

[3] Hopkins L., Pedwell G. (2021). The COVID PIVOT-Re-orienting child and youth mental health care in the light of pandemic restrictions. Psychiatr. Q..

[4] Sullivan A.D.W., Forehand R., Acosta J., Parent J., Comer J.S., Loiselle R., Jones D.J. (2021). COVID-19 and the acceleration of behavioral parent training telehealth: Current status and future directions. Cogn. Behav. Pract..

[5] Agazzi H., Hayford H., Thomas N.J., Ortiz C., Salinas-Miranda A.A. (2021). A nonrandomized trial of a behavioral parent training intervention for parents with children with challenging behaviors: In-person versus Internet-HOT DOCS. Clin. Child Psychol. Psychiatry.

[6] Canário C., Abreu-Lima I., Santos S., Silva-Martins M., Campos J., Enes Rodrigues C., Tavares M., Mansilha H., Torres S., Serra Lemos M. (2021). Delivering Group Lifestyle Triple P through digital practice: A case study with Portuguese parents. J. Fam. Ther..

[7] Wade S.L., Gies L.M., Fisher A.P., Moscato E.L., Adlam A., Bardoni A., Corti C., Limond J., Modi A.C., Raj S.P. (2020). Telepsychotherapy with children and families: Lessons gleaned from two decades of translational research. J. Psychother. Integr..

[8] McAloon J., de la Poer Beresford K. (2021). Online behavioral parenting interventions for disruptive behavioral disorders: A PRISMA based systematic review of clinical trials. Child Psychiatry Hum. Dev..

[9] Breitenstein S.M., Gross D.A., Christophersen R. (2014). Digital delivery methods of parenting training interventions: A systematic review. Worldviews Evid.-Based Nurs..

[10] Harris M., Andrews K., Gonzalez A., Prime H., Atkinson L. (2020). Technology-assisted parenting interventions for families experiencing social disadvantage: A meta-analysis. Prev. Sci..

[11] MacDonell K.W., Prinz R.J. (2017). A review of technology-based youth and family-focused interventions. Clin. Child Fam. Psychol. Rev..

[12] Sanders M.R., Baker S., Turner K.M.T. (2012). A randomized controlled trial evaluating the efficacy of Triple P Online with parents of children with early-onset conduct problems. Behav. Res. Ther..

[13] Lefever J.B., Bigelow K.M., Carta J.J., Borkowski J.G., Grandfield E.M., McCune L., Irvin D.W., Warren S.F. (2017). Long-term impact of a cell phone–enhanced parenting intervention. Child Maltreat..

[14] Van der Zanden R.A.P., Speetjens P.A.M., Arntz K.S.E., Onrust S. (2010). Online group course for parents with mental illness: Development and pilot study. J. Med. Internet Res..

[15] Manav A.I., Gozuyesil E., Tar E. (2021). The effects of the parenting education performed through WhatsApp on the level of maternal-paternal and infant attachment in Turkey. J. Pediatr. Nurs..

[16] Cardamone-Breen M.C., Jorm A.F., Lawrence K.A., Rapee R.M., Mackinnon A., Yap M.B.H. (2018). A single-session, web-based parenting intervention to prevent adolescent depression and anxiety disorders: Randomized controlled trial. J. Med. Internet Res..

[17] Comer J.S., Furr J.M., Miguel E., Cooper-Vince C.E., Carpenter A.L., Elkins R.M., Kerns C.E., Cornacchio D., Chou T., Coxe S. (2017). Remotely delivering real-time parent training to the home: An initial randomized trial of internet-delivered Parent-Child Interaction Therapy (I-PCIT). J. Consult. Clin. Psychol..

[18] Corralejo S.M., Domenech Rodríguez M.M. (2018). Technology in parenting programs: A systematic review of existing interventions. J. Child Fam. Stud..

[19] Fulgoni C.M.F., Melvin G.A., Jorm A.F., Lawrence K.A., Yap M.B.H. (2019). The Therapist-assisted Online Parenting Strategies (TOPS) program for parents of adolescents with clinical anxiety or depression: Development and feasibility pilot. Internet Interv..

[20] Nieuwboer C.C., Fukkink R.G., Hermanns J.M.A. (2013). Online programs as tools to improve parenting: A meta-analytic review. Child. Youth Serv. Rev..

[21] Thongseiratch T., Leijten P., Melendez-Torres G.J. (2020). Online parent programs for children’s behavioral problems: A meta-analytic review. Eur. Child Adolesc. Psychiatry.

[22] Metcalfe R.E., Matulis J.M., Cheng Y., Stormshak E.A. (2021). Therapeutic alliance as a predictor of behavioral outcomes in a relationally focused, family-centered telehealth intervention. J. Marital Fam. Ther..

[23] Owen N. (2020). Feasibility and acceptability of using telehealth for early intervention parent counselling. Adv. Ment. Health.

[24] Nieuwboer C., Fukkink R., Hermanns J.M.A. (2015). Single session email consultation for parents: An evaluation of its effect on empowerment. Br. J. Guid. Couns..

[25] Morawska A., Tometzki H., Sanders M.R. (2014). An evaluation of the efficacy of a Triple P-Positive Parenting Program podcast series. J. Dev. Behav. Pediatr..

[26] EurofamNet. https://eurofamnet.eu/.

[27] Devaney C., Christiansen Ø., Holzer J., MacDonald M., Matias M., Piessens A., Salamon E. The Conceptualisation and Delivery of Family Support in Europe: A Review of Academic Literature. https://eurofamnet.eu/system/files/the_conceptualisation_and_delivery_of_family_support_in_europe_0.pdf.

[28] Dolan P., Zegarac N., Arsić J. (2020). Family support as a right of the child. Soc. Work Soc. Sci. Rev..

[29] Zegarac N., Isakov A.B., Nunes C., Antunes A.P. (2021). Workforce skills in family support: A systematic review. Res. Soc. Work Pract..

[30] Daly M., Bray R., Bruckhauf Z., Byrne J., Margaria A., Pećnik N., Samms-Vaughan M. (2015). Family and Parenting Support: Policy and Provision in a Global Context.

[31] Breitenstein S.M., Fogg L., Fogg L., Fogg L., Ocampo E.V., Acosta D.I., Gross D.A. (2016). Parent use and efficacy of a self-administered, tablet-based parent training intervention: A randomized controlled trial. JMIR Ment. Health.

[32] Enebrink P., Högström J., Forster M., Ghaderi A. (2012). Internet-based parent management training: A randomized controlled study. Behav. Res. Ther..

[33] Sanders M.R., Dittman C.K., Farruggia S.P., Keown L.J. (2014). A Comparison of online versus workbook delivery of a self-help positive parenting program. J. Prim. Prev..

[34] Sourander A., McGrath P.J., Ristkari T., Cunningham C.E., Huttunen J., Lingley-Pottie P., Hinkka-Yli-Salomäki S., Kinnunen M., Vuorio J., Sinokki A. (2016). Internet-assisted parent training intervention for disruptive behavior in 4-year-old children: A randomized clinical trial. JAMA Psychiatry.

[35] Dowling C.B., Smith Slep A.M., O’Leary S.G. (2009). Understanding preemptive parenting: Relations with toddlers’ misbehavior, overreactive and lax discipline, and praise. J. Clin. Child Psychol..

[36] Kaehler L.A., Jacobs M., Jones D.J. (2016). Distilling common history and practice elements to inform dissemination: Hanf-Model BPT programs as an example. Clin. Child Fam. Psychol. Rev..

[37] Bai G., Wang Y., Yang L., Niu W. (2015). Effectiveness of a focused, brief psychoeducation program for parents of ADHD children: Improvement of medication adherence and symptoms. Neuropsychiatr. Dis. Treat..

[38] Vismara L.A., McCormick C., Young G.S., Nadhan A., Monlux K. (2013). Preliminary findings of a telehealth approach to parent training in autism. J. Autism Dev. Disord..

[39] Familias en Positivo. http://www.familiasenpositivo.org/.

[40] NSPCC Support for Parents. https://www.nspcc.org.uk/keeping-children-safe/support-for-parents/.

[41] Facebook Page Parenting for Lifelong Health. https://www.facebook.com/ParentingLH/.

[42] UNICEF ECARO “Parent Buddy”. https://www.unicef.org/eca/media/14796/file.

[43] Jones S., Jovanoska J., Calam R., Wainwright L., Vincent H., Asar O., Diggle P.J., Parker R., Long R., Sanders M.R. (2017). Web-based integrated bipolar parenting intervention for parents with bipolar disorder: A randomised controlled pilot trial. J. Child Psychol. Psychiatry.

[44] Jones D.J., Loiselle R., Zachary C., Georgeson A.R., Highlander A., Turner P., Youngstrom J.K., Khavjou O., Anton M.T., Anton M.T. (2021). Optimizing engagement in behavioral parent training: Progress toward a technology-enhanced treatment model. Behav. Ther..

[45] Kling Å., Forster M., Sundell K., Melin L. (2010). A randomized controlled effectiveness trial of parent management training with varying degrees of therapist support. Behav. Ther..

[46] Suárez A., Byrne S., Rodrigo M.J. (2018). Effectiveness of a universal web-based parenting program to promote positive parenting: Patterns and predictors on program satisfaction. J. Child Fam. Stud..

[47] Spencer C.M., Topham G.L., King E.L. (2020). Do online parenting programs create change?: A meta-analysis. J. Fam. Psychol..

[48] Döpfner M., Wähnke L., Klemp M.T., Mühlenmeister J., Schürmann S., Hellmich M., Plück J. (2020). Efficacy of web-assisted self-help for parents of children with ADHD (WASH)—A three-arm randomized trial under field/routine care conditions in Germa-ny. BMC Psychiatry.

[49] Rozchodem Rodina Nekončí: Podpůrný Program pro Rozcházející se Rodiče a Jejich Děti. https://aperio.cz/portfolio/rozchodem-rodina-nekonci/.

[50] Rozchodem Rodina Nekončí. https://aperio.thinkific.com/courses/rozchodem-rodina-nekonci.

[51] West F., Sanders M.R., Cleghorn G.J., Davies P.S.W. (2010). Randomised clinical trial of a family-based lifestyle intervention for childhood obesity involving parents as the exclusive agents of change. Behav. Res. Ther..

[52] Growing up Together–Online. https://www.rastimozajedno.hr/rastimo-zajedno-online/o-radionicama-rastimo-zajedno-online.

[53] Growing up Together in a New Family. https://www.rastimozajedno.hr/rastimo-zajedno-u-novoj-obitelji/.

[54] Provenzi L., Grumi S., Gardani A., Aramini V., Dargenio E., NAbobi C., Vacchini V., Borgatti R., Engaging with Families through On-line Rehabilitation for Children during the Emergency (EnFORCE) Group (2021). Italian parents welcomed a telehealth family-centred rehabilitation programme for children with disability during COVID-19 lockdown. Acta Paediatr..

[55] Leana-Tascilar M.Z., Ozyaprak M., Yilmaz O. (2021). An online training programfor gifted children’s parents in Turkey. Eurasian J. Educ. Res..

[56] Porozmawiajmy o Rodzicielstwie i Nastolatkach–Bezpłatna Grupa Wsparcia Online. https://stopuzaleznieniom.pl/organizacja/fundacja-studio-psychologii-zdrowia/porozmawiajmy-o-rodzicielstwie-i-nastolatkach-bezplatna-grupa-wsparcia-online/.

[57] Jak Mądrze Wspierać Nastolatka–Grupa Wsparcia Online dla Rodziców Nastolatków w Kryzysach Psychicznych. http://tpm.org.pl/event/jak-madrze-wspierac-nastolatka-grupa-wsparcia-online-dla-rodzicow-nastolatkow-w-kryzysach-psychicznych/.

[58] Clarke J., Proudfoot J., Proudfoot J., Whitton A.E., Birch M.-R., Boyd M., Parker G., Manicavasagar V., Hadzi-Pavlovic D., Fogarty A.S. (2016). Therapeutic alliance with a fully automated mobile phone and web-based intervention: Secondary analysis of a randomized controlled trial. JMIR Ment. Health.

[59] Newman M.G., Szkodny L.E., Llera S.J., Przeworski A. (2011). A review of technology-assisted self-help and minimal contact therapies for anxiety and depression: Is human contact necessary for therapeutic efficacy?. Clin. Psychol. Rev..

[60] García-Peñalvo F.J., Figuerola C.G., Merlo J.A. (2010). Open knowledge: Challenges and facts. Online Inf. Rev..

[61] Prescott J., Rathbone A.L., Brown G. (2020). Online peer to peer support: Qualitative analysis of UK and US Open Mental Health Facebook groups. Digit. Health.

[62] Nieuwboer C.C., Fukkink R.G., Riva G., Wiederhold B.K. (2016). Peer and professional support for parents. The psychology of Social Networking: Personal Experience in Online Communities.

[63] Ali K., Farrer L., Gulliver A., Griffiths K.M. (2015). Online peer-to-peer support for young people with mental health problems: A systematic review. JMIR Ment. Health.

[64] Strand M., Eng L.S., Gammon D. (2020). Combining online and offline peer support groups in community mental health care settings: A qualitative study of service users’ experiences. Int. J. Ment. Health Syst..

[65] UK Facebook Group Tots Together. https://www.facebook.com/totstogether.

[66] Naslund J.A., Aschbrenner K.A., Marsch L.A., Bartels S.J. (2016). The future of mental health care: Peer-to-peer support and social media. Epidemiol. Psychiatr. Sci..

[67] Chan J.K., Farrer L., Gulliver A., Bennett K., Griffiths K.M. (2016). University students’ views on the perceived benefits and drawbacks of seeking help for mental health problems on the internet: A qualitative study. JMIR Hum. Factors.

[68] Barak A., Boniel-Nissim M., Suler J. (2008). Fostering empowerment in online support groups. Comput. Hum. Behav..

[69] Zhang M.W.B., Tran B.X., Le H.T., Nguyen H.D., Nguyen C.T., Tran T.D., Latkin C.A., Ho R.C.M. (2017). Perceptions of health-related information on Facebook: Cross-sectional study among Vietnamese youths. J. Med. Res..

[70] Frish Y., Greenbaum D. (2017). Is social media a cesspool of misinformation? Clearing a path for patient-friendly safe spaces online. Am. J. Bioeth..

[71] Huh J., Marmor R.A., Jiang X. (2016). Lessons learned for online health community moderator roles: A mixed-methods study of moderators resigning from WebMD communities. J. Med. Internet Res..

[72] Kim J.-E., Weinstein E., Selman R.L. (2017). Romantic relationship advice from anonymous online helpers: The peer support adolescents exchange. Youth Soc..

[73] ICT4D–Technology for Children, Young People and Families. https://www.sos-childrensvillages.org/ict4d.

[74] Cole D.A., Nick E.A., Zelkowitz R.L., Roeder K., Spinelli T. (2017). Online social support for young people: Does it recapitulate in-person social support; can it help?. Comput. Hum. Behav..

[75] Trepte S., Dienlin T., Reinecke L. (2015). Influence of social support received in online and offline contexts on satisfaction with social support and satisfaction with life: A longitudinal study. Media Psychol..

[76] DeRubeis R.J., Cohen Z.D., Forand N.R., Fournier J.C., Gelfand L.A., Lorenzo-Luaces L. (2014). The Personalized Advantage Index: Translating research on prediction into individualized treatment recommendations. A Demonstration. PLoS ONE.

